# The Home Hospitals Association for Paying Patients

**Published:** 1911-04-08

**Authors:** 


### THE HOME HOSPITALS ASSOCIATION FOR PAYING PATIENTS.

### THE OPPORTUNITY TO SET UP A PERFECT SYSTEM OF MEDICAL ATTENDANCE.

The thirty-third annual meeting of this Association was held on Friday, March 24.

The Chairman (Sir Henry C. Burdett, K.C.B., K.C.V.O.), read a letter which had been sent by the Duke of Northumberland asking that his Grace's apologies might be tendered to the Committee for his inability to take the chair, as he had been suffering from a severe attack of gout and he found that any over-exertion put him back in health.

#### The Year's Work at Fitzroy House.

The preliminary formalities having been disposed of,

Sir Henry Burdett said it was now his pleasure to propose that the report submitted to the meeting be taken as read and adopted. Those who were familiar with the work of the Home Hospitals Association would be aware that it was the pioneer Nursing Home in this country, and that for thirty-three years it had continued its beneficent work with ever-increasing success. He was much struck with the excellent table of particulars which appeared in the report, for it gave in a nutshell the key to the position at Fitzroy House, a state of affairs of which everyone connected with the institution had reason to feel proud. In his speech at the meeting last year he said it was remarkable that the average stay of each patient at Fitzroy House was only seventeen days, whereas the average stay, in the best hospitals, of acute surgical cases, though it had been reduced from thirty-five days, still stood at about twenty-one days. This year the average stay at Fitzroy House had been reduced to sixteen days. In a measure, that might be due to the admission of a larger number of cases which were dealt with quickly and recovered rapidly; but even allowing for such, he maintained that the average stay spoke volumes for the hygienic condition and the good management of the Association. There were two other points worthy of remark. The admissions during last year numbered 449, the largest in any year in the House, and the income had also reached a record figure, £8,529. On those two facts he heartily congratulated everybody connected with the Home. Seventy-eight members of the medical profession had found in the Home everything they required when they needed hospital care for their private patients before and after operation. Another fact which spoke volumes for the attention, care, and excellence of management of the Committee was the supreme control, from the earliest days to the present time, of Mr. Frederick Cox. When the hospital was rebuilt in 1902, there was a great difficulty about raising the funds necessary to defray the expense, amounting to many thousands of pounds. In consequence of that, the Duke of Northumberland, Mr. Cox, and himself incurred somewhat onerous pecuniary liabilities. He was glad to note that with this year's report their liabilities ceased, because owing to the careful management the sum for which they had been responsible had been earned and repaid. He was sure he could express, not only on his own behalf and on that of the Governors, but also on behalf of the patients who owed so much to Fitzroy House, their warm appreciation of the excellence of the Home and its administration. He also took it as evidence that hospitals for paying patients were very earnestly desired by large and increasing numbers of the population. And it was a very remarkable, as well as a regrettable, fact that though they had been working there for over thirty years as the pioneer institution, they had not yet succeeded in getting that urgent and much-needed reform introduced into the system of hospital relief. He wished further to say that the success of the Home had been very largely due, as the medical gentlemen who had used it would agree, to the able management of Miss Pearson, and the absolute satisfaction and confidence which the medical staff reposed not only in Miss Pearson, but in every nurse and official of the hospital, a confidence which was shared by the patients, who were very grateful for the care and attention they received.

#### The Future of Hospitals.

Last year he spoke about the future of the hospitals, and that address, which attracted a good deal of attention, had led to important movements, which had not yet fructified, or been made public, in the direction of a total rearrangement of the present system of medical relief. In regard to the question of medical treatment for all classes, the opportunity of the moment was one which should be grasped and welcomed. It afforded the ready introduction of a perfected, up-to-date, exhaustive, and complete medical hospital and nursing service, providing for all classes, reserving throughout the vital principles of thrift and self-reliance, with a maximum of efficiency. Such a service, which when organised would embrace and bring into working co-operation every existing agency for medical relief, however supported and however administered, would at once render an immense service to humanity by lessening the sufferings caused to masses of the population in towns, owing to their ignorance and want of opportunity to learn how to find exactly the relief they required, as and when they required it. The keystone to success was co-operation. At the present time there was a good deal of overlapping between the Poor Law and the voluntary systems of medical relief. He would like to carry the point a little further. If a great voluntary hospital desired to prevent abuse and to enforce a system whereby abuse should be avoided, though the best interests of the patient should always be safeguarded, it was met with difficulties, from whichever direction it approached the question. A patient applied to the hospital, and was admitted. It appeared that his circumstances were such that he could well afford to pay a reasonable sum for his maintenance in the hospital. But the hospital authorities were at once in the difficulty, that medically it might be inhuman to send him out, but practically they had no paying ward to which they could transfer him. In the out-patient department one could find a large number of patients—though he thought that, on the whole, the out-patient department was probably less abused materially than the in-patient department—whose ailments were not severe, and which could be as well, or perhaps better, attended to by a private medical attendant or a provident institution. At the present time there was no system of co-operation or intercommunication, and no means of transferring the patient from the hospital to the doctor or agency which ought, properly, to undertake his relief and care. He believed that the one remedy for the ever-increasing number of patients who flocked to the out-patient departments of the hospitals, largely to their own undoing, was that the hospitals should agree to abolish the out-patient department altogether. (Hear, hear.)

### Reform of Sick Relief.

There was no doubt that in cities the means of medical relief of all kinds were sufficiently great to enable everybody to be provided for frequently and adequately, in accordance with their means. It was that fact which made him so insistent that the present moment should be taken advantage of, in dealing with the Poor Law to deal with the whole question of medical relief, and to bring everything into co-ordination and into co-operation. The out-patient department should be abolished, and all applicants for out-relief passed through the casualty department, which every hospital must have. Every case should be treated there once; then the patients should be sorted and sent some to the private practitioner, some to the provident dispensary, or, in the cases of severer nature, should be brought up to the consultation department of the great hospitals. These latter cases should not be hurriedly rushed through in hundreds, but treated where there was abundance of time for the best brains and skill of the honorary medical staff to deal with each case upon its merits, and do the best for the patient. That was a very simple adjustment administratively, though it was a great revolution in regard to medical relief as it existed to-day. Without injury to anyone, it would effect great benefit, and become immensely popular so soon as it got thoroughly into working. He did not believe that the time occupied in introducing such a system would be more than from six to twelve months; and the change would result in enormous benefit, not only by increasing the comfort of the conditions of treatment which hospitals were well able to offer to all patients, but by giving every patient an opportunity to exhibit that thrift and self-dependence which were so sadly needed at the present day in this country. As to the in-patient department, the reform there was one which would also embrace the Poor Law institutions, and might be adapted with ready facility. England should take an example from the Continent, and from the United States. There the hospitals classified their wards. They had wards commencing with those in which the patients paid a remunerative rate for the relief and treatment which they received. They had two other classes, in one of which the patient paid an inclusive rate which covered all the expenses of the medical charges, and in the other the medical attendance was unpaid for, but the whole of the maintenance charges were paid. There was also a fourth class, in which what we knew as Poor Law cases which were actually ill would be included, and were paid for by the municipality, or, in our case, by the County or Municipal Council, or whatever the local authority might be which was responsible for the maintenance of and proper provision for the sick. That suggestion appealed very strongly to the gentlemen who devoted so much time and attention to the work of the Poor Law Commission; and as a result of that, he, Sir Henry, issued a statement entitled "The Future of the Hospitals"; and many gentlemen had been considering the question of the entire remodelling and reform of the whole of our system of medical relief, and good progress was being made. He hoped that before many years had passed these ideas would find expression in an Act of Parliament, which would thus unite every available force for overcoming disease and dealing with accidents, in a bond of workers who would never overlap, so that the whole energy they possessed might be brought to bear upon the work, whilst every farthing of money voted for the purposes of medical relief would be available to be devoted entirely to the object for which it had been provided, and that money would be free from the abuse of overlapping which at present reduced and crippled the resources of medical agencies of all kinds throughout the kingdom.

### The Government Scheme.

Government action had been delayed by the political situation. There was no prospect or opportunity for any movement in the direction of a Bill for the reform of the Poor Law system, or the remodelling of medical relief during the present session. They were, however, awaiting with interest the coming Budget, because it had been intimated that the Chancellor of the Exchequer proposed to provide for the introduction of a great system of insurance, which should be coupled with the Old Age Pension system that had recently been sanctioned by Parliament, with such momentous results for the good of the older and poorer members of the race. He attached very great importance to the Budget, because if the proposal of insurance was to follow the practical lines which had been adopted with such wonderful success in Germany, for instance, or in some of our Colonies, no doubt it would be capable of being made available also to assist in the remodelling and codification and unity of all systems and agencies for the relief of the sick in this country.

### The Home Dangers of Modern War.

He had been much abroad during the past year, and in discussing the question with some very able public men in Germany he found that the effect of that closer attention to the just needs of the poorer citizens of a great country—those of the workers, who were the backbone of the country—was such as to defeat, before long, the threatened dangers of an Armageddon, which so many thinking men of all countries were coming to dread as the ever-increasing thirst and competition for armaments went on. One gentleman, who knew well what he was talking about, and had studied the question, said that although Germany possessed such an enormous fleet, and though she was maintaining probably the greatest and the best-found army the world had ever had, yet it was not within the power of the rulers of Germany, as represented by those responsible for its government, to make war at their will. He maintained—and it seemed to be well-known in Germany as a fact—that if the army of Germany were to be withdrawn from the country in order to wage war outside its boundaries, there was enormous danger that the cities of Germany might even be controlled and occupied by the people who would not have war if they could possibly avoid it, and who, for political and other reasons, might take advantage of the absence of the troops to make their power felt and their will prevail if war should be entered upon without their full concurrence and consent. That was a position which might startle people who did not think about those questions, but behind the agitation for arbitration and co-operation among the nations to prevent war there was a strong and ever-growing and increasing body of public opinion, which was not confined to any class of citizens, which was making its way with most people. He did not believe war could be stopped by arbitration, but he did believe that, whereas it was necessary for every nation to be armed and capable of defending its possessions, that social legislation in the direction of insurance and old-age provision, and other matters, was tending directly against war, was uniting people more and more to hate war; and he was hopeful that the time might yet come when wars, if they would not cease, would yet be so rare as to be remarkable.

Movements Towards Co-operation.

Although there was no prospect of Government action at present in regard to medical relief for all classes, there was independent action going on which was of very great importance. The Prince of Wales, when he was President of King Edward's Hospital Fund promised, at the last annual meeting, that an Out-Patient Commission should be appointed to go into that question fully and authoritatively. Lord Mersey, Lord Northcote, and the Bishop of Stepney were appointed a Commission, and they were now sitting and conducting a very careful and exhaustive inquiry. He hoped that one result of that inquiry would be a recommendation that, in the best interests of all concerned in the matter, the out-patient department of the hospitals should be abolished, and that in substitution for them there might be a casualty department, and that the plan which he outlined at the commencement of his speech might be generally followed by all the great hospitals of the country. As to the Poor Law Commission reports, it was only right that the public should understand that a great deal of work had been done in connection with these reports. Mr. John Burns, he felt confident—though he had no authority for the statement—had been continuously studying these reports, so that when the proper moment offered, one might anticipate suggestions which would be fruitful in good results.

Sunday Shows and Hospitals.

While dealing with hospitals, he would like to mention that there was one other question which had attracted some attention, and was likely to lead to some difficulty, namely, that of cinematograph shows and the Hospital Sunday Fund. Side-shows, as exhibition managers knew, were a chief source of revenue. But side-shows as represented by the Sunday cinematograph exhibitions in connection with Hospital Sunday might, if great care and tact were not used, result in the loss of a very large income to the voluntary hospitals. He had been abroad and returned only a few days ago, and was then astonished to find how deep and extended was the feeling, on one side of hostility to the opening of cinematograph shows on Sunday; and, on the other side, on the part of those who thought—and he was one of those—that it was a good thing and a necessary thing in cities to give the population every opportunity possible to have places of resort where they could spend their leisure, which would at the same time protect them from the dangers of the streets and the discomforts, miseries, and dangers of the public-house, and provide relaxation apart from the very limited accommodation at home. However that might be, the Committee of the Hospital Sunday Fund would have before it the decision of the problem, and upon the result of its decision and the wisdom of it might possibly depend the continuance of the Hospital Sunday collections. The situation in regard to the Sunday cinematograph shows had arisen entirely from the action of a few hospitals. The London County Council only permitted cinematograph shows to be opened on Sunday when it had received a request from the managers of a hospital or some other charity for permission to hold such show on the Sabbath in the interest of such charity. The results of that exploitation of a new source of revenue, which every hospital manager in London was quite at liberty to avail himself of if he thought fit, had been to produce, in the case of one hospital, a revenue of about £1,400 a year. There were only altogether about seven hospitals which had taken part in that kind of business, and on them rested the responsibility for the whole of the difficulty which had arisen. On the one hand, the Hospital Sunday Fund must take care to keep itself free from any expression of opinion as to the merits of the question of Sunday cinematograph exhibitions, and must leave a free hand to the hospitals to raise their income from whatever source they might think it best and desirable in their own interests. But a free hand must also be left to the congregations who sent their collections to the Mansion House for distribution by the Sunday Fund, regarding that Fund as a trustee in the interests of the congregation and also in the interests of the sick.

The Case Against Action.

The possible effects of that Sunday cinematograph controversy, if it were not wisely dealt with, could be put into a nutshell. On the one hand, the total possible revenue from the cinematograph shows on Sunday for hospitals was between £4,000 and £5,000 a year. The Hospital Sunday collections in London from the churches amounted to £40,000 a year. Thus there was the danger that in the justifiable endeavour to increase the income of certain hospitals to the extent of some £5,000 a year they — perhaps innocently and unintentionally — were face to face with the possibility that the Hospital Sunday Fund might be irretrievably damaged. Taking the whole of the hospitals of the country, the Sunday Fund contributed 6 per cent. of the total income of the best hospitals, or £165,000 a year. That was taking 814 hospitals. The ordinary income of the hospitals at the present time, according to the latest figures, was two and three-quarter millions, *plus* three-quarters of a million from legacies, and nearly £600,000 from special donations and building funds. That made a total income for 1908 of £4,052,000 in the case of those 814 hospitals, against an expenditure of £3,701,000. That gave a surplus of £351,000 a year for the whole of those hospitals which were conducted under the voluntary system. He always liked to give those figures year by year, in order to show that those who were pessimists and held that the voluntary system was dying and was inadequate, had no grounds on which to base their fears. If there were a system of co-operation which prevented overlapping, a very large sum would be made available for the development of methods of treatment, the extension of methods of research, and for use in many other ways, which would tend to make the hospital as an up-to-date agency for the relief and cure of disease even more efficient than it was at the present time. But the loss of £160,000 a year from Hospital Sunday would grievously cripple voluntary hospitals. In effect it would adversely affect the voluntary hospital revenue of the whole country to an extent equal to the immense good done to the revenue of the London hospitals by the King's Hospital Fund. That was one way of showing the risks of the present situation, and he did hope that those who were responsible for the decision as to the policy to be pursued by the Council of the Hospital Sunday Fund concerning the opening of cinematograph shows on Sunday, would look at the question all round, and pause before they committed themselves to a course which had definite dangers ahead.

The Fruits of Co-operation.

At present the voluntary hospitals possessed considerable reserves, but, as he had said, the system of co-operation and the unification of all existing agencies for medical treatment and cure would increase those reserves at once to a large amount. Therefore, he contended that every public-spirited man and woman should make it his or her business to work for co-operation in that sense. Then Great Britain would speedily secure the best system and the most perfect system of medical relief which the world had ever seen. He hoped he had not occupied too much time, but he believed it was very important on those questions, as they were now at the parting of the ways, that they should take the opportunity afforded by a meeting of that kind—using as a platform this hospital which had been such a marked and progressive success—to drive home certain truths which were well worthy of public attention, and which stood in need of much reiteration before there could be won for them that measure of attention which, on their merits, they undoubtedly deserved.

